# Evolutionary divergence in tail regeneration between *Xenopus laevis* and *Xenopus tropicalis*

**DOI:** 10.1186/s13578-021-00582-9

**Published:** 2021-04-07

**Authors:** Shouhong Wang, Yun-Bo Shi

**Affiliations:** Section on Molecular Morphogenesis, Eunice Kennedy Shriver National Institute of Child Health and Human Development (NICHD), National Institutes of Health (NIH), Bethesda, MD USA

**Keywords:** *Xenopus laevis*, *Xenopus tropicalis*, Tail regeneration, Refractory period, Evolution

## Abstract

Tissue regeneration is of fast growing importance in the development of biomedicine, particularly organ replacement therapies. Unfortunately, many human organs cannot regenerate. Anuran *Xenopus laevis* has been used as a model to study regeneration as many tadpole organs can regenerate. In particular, the tail, which consists of many axial and paraxial tissues, such as spinal cord, dorsal aorta and muscle, commonly present in vertebrates, can fully regenerate when amputated at late embryonic stages and most of the tadpole stages. Interestingly, between stage 45 when feeding begins to stage 47, the *Xenopus laevis* tail cannot regenerate after amputation. This period, termed “refractory period”, has been known for about 20 years. The underlying molecular and genetic basis is unclear in part due to the difficult to carry out genetic studies in this pseudo-tetraploid species. Here we compared tail regeneration between *Xenopus laevis* and the highly related diploid anuran *Xenopus tropicalis* and found surprisingly that *Xenopus tropicalis* lacks the refractory period. Further molecular and genetic studies, more feasible in this diploid species, should reveal the basis for this evolutionary divergence in tail regeneration between two related species and facilitate the understanding how tissue regenerative capacity is controlled, thus with important implications for human regenerative medicine.

**Supplementary Information:**

The online version contains supplementary material available at 10.1186/s13578-021-00582-9.

## Dear Editor

Tissue regeneration is essential for the homeostasis and function of a number of mammalian organs, particularly those exposed to external environment, such as hair, skin, and intestine. Unfortunately, for most other mammalian organs, the capacity to heal wounds and regenerate is poor and decreases with age. On the other hand, many organisms across the animal kingdom, such as hydra, zebrafish and amphibians, have remarkable abilities to regenerate body parts following traumatic injury [1]. Among them, the anuran amphibian *Xenopus (X.) laevis* has been a powerful model for studying regeneration. For example, its tail could regenerate completely, including the axial and paraxial tissues, such as spinal cord, notochord, dorsal aorta and muscle, following amputation from the early tailbud to metamorphic stages. Interestingly, this regenerative ability is transiently lost around the onset of feeding (stages 45–47), referred to the “refractory period” [2]. Extensive studies have been carried out to understand the regeneration mechanism including why the refractory period is present [3, 4]. For example, earlier studies have found that at the cellular level, there are a failure of ROCs (regeneration-organizing cells) to migrate to the amputation site and a presence of a high level of apoptosis at the refractory stages [3, 5]. In addition, inappropriate activation of the immune system may also impair tail regeneration, as immune suppression improves regeneration in these animals [5]. However, the underlying molecular and genetic basis for the refractory period remains unknown.

**Fig. 1:**
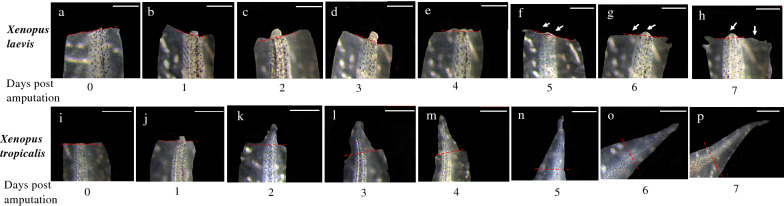
*X. tropicalis* but not *X. laevis* tadpoles at stage 46 can regenerate its tail after amputation. **a**–**h** *X. laevis* tail at 0–7 days, respecitvely, after amputation at stage 46. The tail did not regenerate, and the cut surface became covered with a thick skin-like epithelium (white arrows). **i**–**p** *X. tropicalis* tail at 0–7 days, respectively, after amputation at stage 46. Note that significant tail regeneration occurred by 2 days (**k**). Scale bar is 0.86 mm. The red dashed line indicates the amputation plane

Recent advancements in genome annotation and gene editing technologies have made the diploid anuran *X. tropicalis* a superior model than the pseudo-tetraploid *X. laevis* for genome-wide and genetic studies. While studying tail regeneration in *X. tropicalis,* we were surprised to observe that tadpoles at stage 46, shortly after feeding begins at stage 45, were able to regenerate the tail after amputation. As shown in Fig. 1, *X. laevis* tadpoles at stage 46, as expected, failed to generate any of the amputated tail even 7 days after amputation, while nearly complete regeneration of the tail was observed by 5–6 days after of amputation for *X. tropicalis* tadpoles at stage 46. Careful examination of the amputated tadpoles showed that for *X. laevis* tadpoles, the tail finished wound healing and formed the epidermis at the amputation site 1 day later (Fig. 1b). However, the tail did not regenerate subsequently even 7 days later, with only a stump at the amputated site (Fig. 1h). Instead, the cut surface became covered with a thick skin-like epithelium (Fig. 1f–h). Interestingly, even when we reared such amputated animals for 2 months, we could only observe a stump at the amputated site (Additional file 1: Fig. S1b). By this time, the tadpoles had developed to metamorphic climax and tail resorption had begun (See Additional file 1: Fig. S1a for an example at stage 63 when most of the tail had resorbed). Subsequently, all animals finished metamorphosis and became froglets without tail regeneration.

For *X. tropicalis* at stage 46, the tail also finished wound healing and formed the epidermis at the amputation site 1 day after amputation (Fig. 1j). However, by 2 days, significant regeneration had occurred (Fig. 1k). Essentially complete regeneration occurred after 5 days with the amputation plane difficult to observed by 7 days (Fig. 1p).

To investigate if the refractory period observed in *X. laevis* is still present in *X. tropicalis* but shifts to a different stages, we examined tail regeneration of *X. tropicalis* animals from tailbud stages (stage 29/30) to the climax of metamorphosis (stages 58/59) and observed regeneration at every stages that we analyzed, suggestion that the refractory period is absent in *X. tropicalis.*

**Fig. 2:**
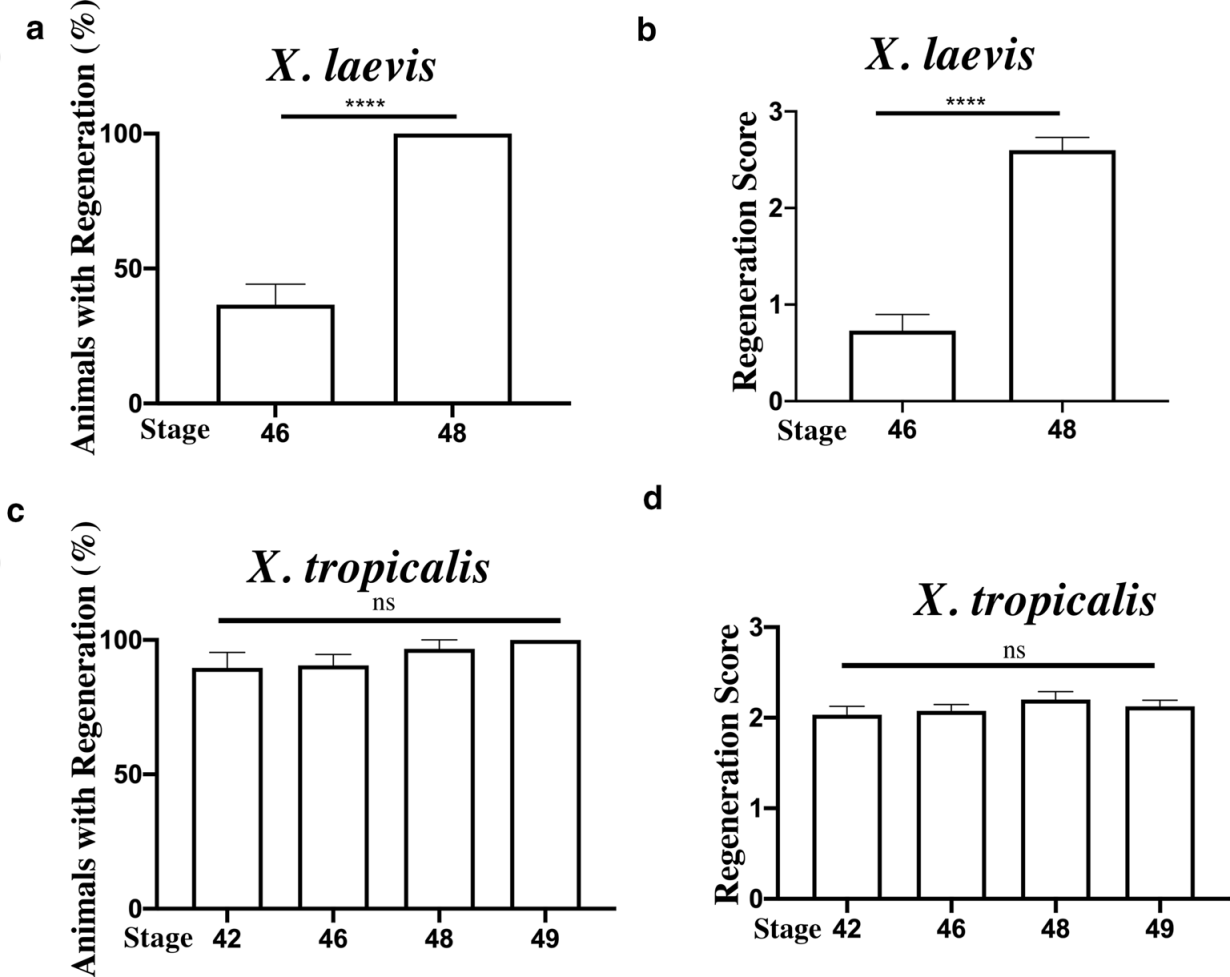
No refractory period in tail regeneration in *X. tropicalis.* **a**, **c** Percent of animals had tail regeneration 7 days after amputation at indicated stages. Note that for *X. laevis,* nearly 100% tadpoles could regenerate the tail when amputated at stage 48 while only 37% could at stage 46. For *X. tropicalis,* nearly 100% could generate at all stages between 42 and 49. **b**, **d** The average regeneration score 7 days after amputation at indicated stages. Note that *X. laevis,* the average regeneration score at stage 46 was under 1, indicating absence of or very poor regeneration, while the score at stage 48 was close to 3, full regeneration. For *X. tropicalis,* the average regeneration score was all above 2 at all stages, indicating good regeneration, and no significance difference was found among different stages*. *ns, non-significant and ****p < 0.0001

To quantitatively compare the regeneration capacities around the refractory period (stages 45–47 in *Xenopus laevis*) between the two species, we determined the percent of animals at stages 42–49 that could regenerate the tail 7 days after amputation and also measured the regeneration index. We assigned a regeneration score based on the morphology of the regenerated tail (Additional file 1: Fig. S2), with 0 as no regeneration and 3 as full regeneration. All animals with a regeneration score of 2 or 3 were counted toward the percent of animals that could regenerate at each stage. As shown in Fig. 2a, for *X. laevis,* only about 37% had some regeneration at stage 46 (a refractory stage), but nearly 100% could regenerate the tail at stage 48 (outside of the refractory period). In addition, the average of regeneration score was less than 1 (poor regeneration) at stage 46 but close to 3 (full regeneration) at stage 48 (Fig. 2b). These results are consistent with earlier findings and confirm the presence of a refractory period in tail regeneration at the start of feeding in *X. laevis* [2].

For *X. tropicalis*, we found that essentially 100% of the tadpole could regenerate after tail amputation at stages 42, 46, 48, 49, encompassing the refractory period of stage 45–47 observed in *X. laevis*, and there was no significant difference among the stages (Fig. 2c). Similarly, average regeneration score was all above 2 for all stages and there was again no significant difference among the different stages (Fig. 2d). These findings indicate that there is no refractory period in tail regeneration in *X. tropicalis*.

*Xenopus laevis* is a powerful model to study regeneration mechanisms [6]. Earlier studies have discovered cellular and molecular differences between regeneration -competent and -incompetent tails. For example, at the cellular level, ROC (regeneration-organizing cell) migration and high levels of apoptosis was observed during the refractory period compared to the regeneration-competent stage [6, 7]. In addition, studies have shown that immune and inflammatory signaling pathways are tightly regulated during tail regeneration [8] and that regeneration can be enabled during the refractory period by activating either BMP or Notch signaling pathways [9]. However, the role of these pathways, particularly the endogenous genes, have not been investigated due to the difficulty for genetic studies in the pseudo-tetraploid organism.

Our data here for the first time report an evolutionary divergence in tail regeneration between two highly related species. The ability of *X. tropicalis* to regenerate its tail now offers an opportunity for studying the function of genes and signaling pathways in regeneration by using gene editing approaches in this diploid organism and for genome wide molecular characterization and discovery approaches because of its annotated genome. The presence and absence of the refractory period in *X. laevis* and *X. tropicalis,* respectively, offers an opportunity for comparative studies to reveal insights on the mechanisms controlling regeneration and for determining whether the refractory period is “lost” in *X. tropicalis* or “gained” in *X. laevis* during evolution. Such studies should help to uncover the molecular and genetic basis for the evolutionary divergence in tail regeneration between these two highly related species and improve our ability to manipulate tissue regeneration for regenerative medicines for human therapies.

## Supplementary Information

**Additional file 1: Figure S1.** A *X. laevis* tadpole amputated at stage 46 failed to regenerate the tail even after two months when the animal reached the metamorphic climax stage 63. **Figure S2.** Different tail regeneration phenotypes observed 7 days after amputation of stage 46 *X. tropicalis* tadpoles.

## Data Availability

All data generated or analysed during this study are included in this published article [and its supplementary files].

## Abbreviations

ROCs: Regeneration-organizing cells
*X. laevis*: *Xenopus laevis*
*X. tropicalis*: *Xenopus tropicalis*
ROS: Reactive oxygen species
BMP: Bone morphogenetic proteins
TGF-*β*: Transforming growth factor*β*
