# Correction to: Evolutionary divergence in tail regeneration between *Xenopus laevis* and *Xenopus tropicalis*

**DOI:** 10.1186/s13578-021-00615-3

**Published:** 2021-06-04

**Authors:** Shouhong Wang, Yun-Bo Shi

**Affiliations:** Section on Molecular Morphogenesis, Eunice Kennedy Shriver National Institute of Child Health and Human Development (NICHD), National Institutes of Health (NIH), Bethesda, MD USA

## Correction to: Cell Biosci (2021) 11: 71 https://doi.org/10.1186/s13578-021–00582-9

The two Additional figures (Additional file 1) were accidentally left out in the linked file during the production of the published version of the paper by Wang and Shi [1].

## Supplementary Information

**Additional file 1: Fig. S1.** A *X. laevis* tadpole amputated at stage 46 failed to regenerate the tail even after two months when the animal reached the metamorphic climax stage 63 and most of the tail was resorbed (a, scale bar is 6.9 mm). The amputated tail tip remained as a stump (b, scale bar is 1.7 mm; the red dashed line indicates the amputation plane). **Fig. S2.** Different tail regeneration phenotypes observed 7 days after amputation of stage 46 *X. tropicalis* tadpoles. (a) “Excellent”: a regenerated tail with an elongation indistinguishable from normal tails, except for missing somite segmentation. (b) “Good”, regenerated tail had defected elongation or lacked fin regeneration. (c) “Partial”, regenerated tail was much shorter, or had defects in patterning and lacked fin regeneration, or had an elongated bulge formation. (d) “None”, the tail had either a blunt end or a small bulge/stump at the amputated site. The regeneration score for the type of tail regeneration in a, b, c, d was assigned 3, 2, 1, 0, respectively. The red dashed line indicates the amputation plane. Scale bar is 1.1 mm.
