# Supplementary material for: Correction to: Evolutionary divergence in tail regeneration between Xenopus laevis and Xenopus tropicalis
Source: Cell Biosci. 2021 Jun 4;11:104. doi: 10.1186/s13578-021-00615-3 (PMC8178875; doi:10.1186/s13578-021-00615-3)
Supplement: Supplementary file 1 — Additional file 1: Fig. S1. A X. laevis tadpole amputated at stage 46 failed to regenerate the tail even after two months when the animal reached the metamorphic climax stage 63 and most of the tail was resorbed (a, scale bar is 6.9 mm). The amputated tail tip remained as a stump (b, scale bar is 1.7 mm; the red dashed line indicates the amputation plane). Fig. S2. Different tail regeneration phenotypes observed 7 days after amputation of stage 46 X. tropicalis tadpoles. (a) “Excellent”: a regenerated tail with an elongation indistinguishable from normal tails, except for missing somite segmentation. (b) “Good”, regenerated tail had defected elongation or lacked fin regeneration. (c) “Partial”, regenerated tail was much shorter, or had defects in patterning and lacked fin regeneration, or had an elongated bulge formation. (d) “None”, the tail had either a blunt end or a small bulge/stump at the amputated site. The regeneration score for the type of tail regeneration in a, b, c, d was assigned 3, 2, 1, 0, respectively. The red dashed line indicates the amputation plane. Scale bar is 1.1 mm. [file 13578_2021_615_MOESM1_ESM.pdf]

Evolutionary divergence in tail regeneration between *Xenopus laevis* and *Xenopus tropicalis*

Shouhong Wang, Yun-Bo Shi*

Section on Molecular Morphogenesis, Eunice Kennedy Shriver National Institute of Child Health and Human Development (NICHD), National Institutes of Health (NIH), Bethesda, Maryland, MD, USA

Additional file 1

Materials and Methods

Experimental animals

Wild type adults *X. tropicalis* and *X. laevis* were purchased from NASCO. Tadpoles were staged according to [1]. Embryos and tadpoles were generated as described [2]. All animal care and treatments were performed as approved by the Animal Use and Care Committee of Eunice Kennedy Shriver National Institute of Child Health and Human Development of the National Institutes of Health.

Amputation procedure

Tadpoles at indicated stages were first anesthetized in 0.02% MS222 (TCI, Tokyo, Japan) in 0.1X MMR (0.1 M NaCl, 2.0 mM KCl, 1 mM $MgSO_4$, 2 mM $CaCl_2$, 5 mM HEPES (pH 7.8). They were transferred to fresh 0.1X MMR before amputation to remove 30-50% (5 mm or less) of the tail by using a sterile scalpel. 30 amputated tadpoles were kept in a tank containing 2 l 0.1X MMR and 50 μg/ml gentamicin. All amputated tadpoles are kept in 25℃ incubators for 7 days with daily 50% water change starting from day 3. Regeneration score for individual tadpoles was assessed at 7 after amputation, with regeneration being excellent, good, partial, or none (Fig. S2), assigned the score of 3, 2, 1, or 0, respectively [3].

Statistical analyses

For quantitative analysis of the regeneration, we determined the regeneration score and percent of animals with regeneration (a tadpole is considered to have regeneration if the regeneration score is 2 or 3). The percent of animals with regeneration was calculated by dividing the number of tadpoles with regeneration with the total number of amputated tadpoles in a sample. The average regeneration score for each sample was also calculated. All statistics were performed by using GraphPad Prism 9. The significance of the differences between two different samples or among four different samples was tested by using Mann–Whitney U-test or ANOVA respectively. Differences were considered significant for $p < 0.05$.

References:

1. Nieuwkoop PD, Faber J. Normal table of *Xenopus laevis.* London:1956.p.170-195.

2. Shibata Y, Tanizaki Y, Shi YB, Thyroid hormone receptor beta is critical for intestinal remodeling during *Xenopus tropicalis* metamorphosis. Cell Biosci. 2020;10:46, 1-15.

3. Aztekin C, Hiscock TW, Marioni JC, Gurdon JB, Simons BD, Jullien J. Identification of a regeneration-organizing cell in the *Xenopus* tail. Science. 2019; 364:653-658.

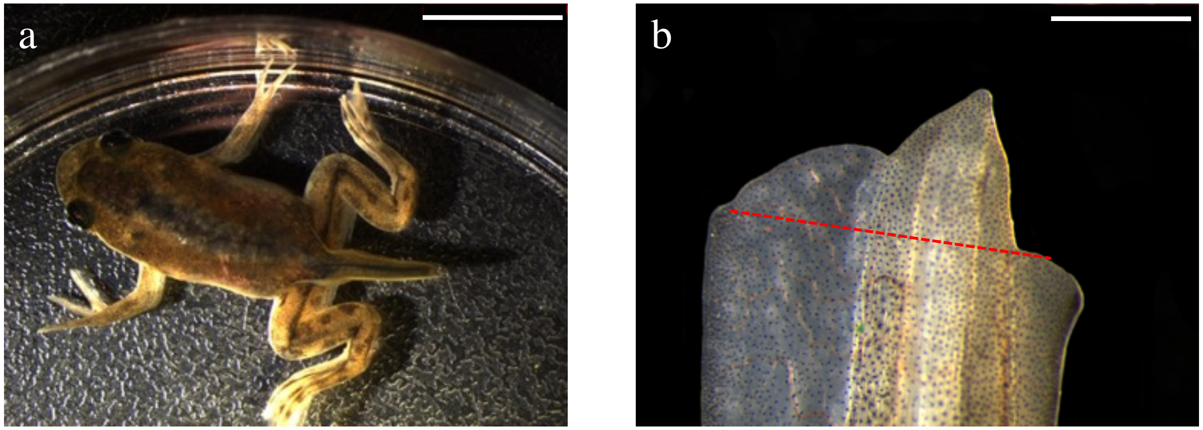

Supplemental Fig. 1. A *X. laevis* tadpole amputated at stage 46 failed to regenerate the tail even after two months when the animal reached the metamorphic climax stage 63 and most of the tail was resorbed (a, scale bar is 6.9 mm). The amputated tail tip remained as a stump (b, scale bar is 1.7 mm; the red dashed line indicates the amputation plane).

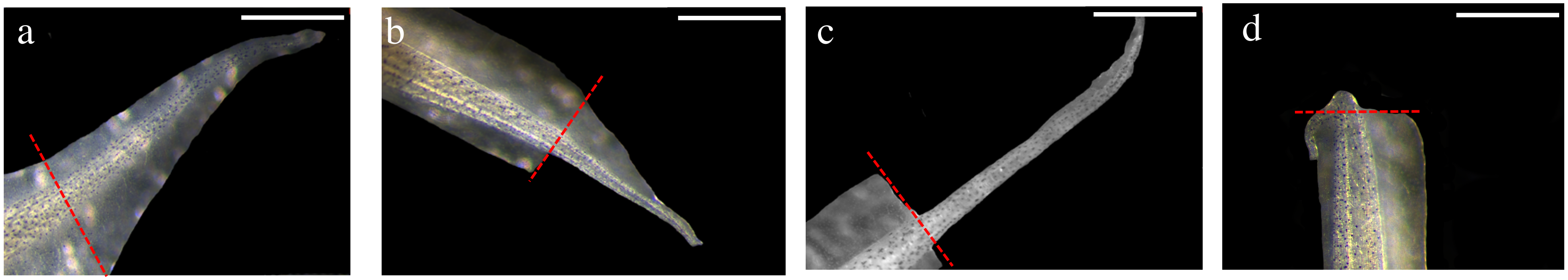

Supplemental Fig. 2. Different tail regeneration phenotypes observed 7 days after amputation of stage 46 *X. tropicalis* tadpoles. (a) “Excellent”: a regenerated tail with an elongation indistinguishable from normal tails, except for missing somite segmentation. (b) “Good”, regenerated tail had defected elongation or lacked fin regeneration. (c) “Partial”, regenerated tail was much shorter, or had defects in patterning and lacked fin regeneration, or had an elongated bulge formation. (d) “None”, the tail had either a blunt end or a small bulge/stump at the amputated site. The regeneration score for the type of tail regeneration in a, b, c, d was assigned 3, 2, 1, 0, respectively. The red dashed line indicates the amputation plane. Scale bar is 1.1 mm.
